# Surgical practice and operative surgical strategies during the COVID-19 pandemic: A commentary

**DOI:** 10.1016/j.amsu.2020.04.046

**Published:** 2020-05-16

**Authors:** Vasileios Karampelias, Ypatios Spanidis, Ioannis Kehagias

**Affiliations:** Department of Surgery, School of Medicine, University of Patras, Patras, 26504, Greece

This article aims to provide updated information regarding appropriate surgical approaches and strategies during the coronavirus-19 (COVID-19) pandemic. The COVID-19 outbreak initially hit China and was transmitted to almost all other countries, resulting to more than 2,4 million infections and 165,000 deaths to date. Hospitals have become hot zones for treatment of this disease, often becoming overwhelmed by individuals infected or suspected of infection, thus increasing the risk of viral transmission. Individuals working in health-care facilities may easily become infected (approximately 15,000 health care workers in Spain, accounting for 14% of the confirmed cases) and the available human resources are decreasing, markedly compromising the effort to prevent the spread and consequences of COVID-19 [1]. Therefore, procedures and examinations should be performed under new strict preventing measures.

Surgical care is considered to be in the frontline of health-care provision in every health-care system. However, the presence of numerous individuals in the operating theaters, the close contact with the patient, and the urgent management have placed surgical procedures in the spotlight during these critical times, as the virus is rapidly transmitted and could potentially infect surgical-care workers. Indeed, to date, there is satisfactory evidence that COVID-19 is mainly transmitted with close contact via respiratory droplets and fomites [2]. Especially, performing aerosol-generating surgical procedures, such as laparoscopy and endoscopy, presents increased infection risk for anesthesiologists, surgeons, and surgical staff attending in the operating room (OR). Although the health-care systems, especially in Western countries, are well organized to deal with such high-risk situations, there are many factors that can influence surgeon decision making and safety, such as the limited available resources and the suffocating pressure due to the increased patient admission. Therefore, the surgeons should consider a number of factors and design clear and effective strategies for the surgical management of patients.

First, it is important for every surgical team to prepare for a rapidly evolving situation that may affect the available resources. Indeed, respirators, facial masks, wipes, and tissues are extensively used during this period, requiring very careful management. Moreover, all human resources must remain committed to critically ill patients. Therefore, each surgical case should be evaluated and elective operations should be postponed to reduce patient traffic in the hospital, the spread of disease, engagement of the health-care staff with patients in pre- or postoperative care, and preserve the availability of hospital beds and protective equipment [3]. According to guidelines from Italy and China, two of the countries most affected by the pandemic, only emergency operations and elective cancer surgeries should be performed, although this strategy has faced opposition and criticism in other countries [4,5]. Likewise, an Italian team suggested postponing all surgical procedures of patients suspected to have COVID-19 until confirmed infection clearance [6]. At this point, we should note that despite the fact that several cancer-related surgeries, such as biopsies, are elective, they are also considered essential and would thus not be prudent to postpone them.

Moreover, the education of staff regarding COVID-19 management and appropriate personal protective equipment (PPE) use is of great importance. The risk is highest when there is lack of awareness regarding specific safety details. For instance, although staff is used to wearing N95 masks, they sometimes do not fit well, which reduces their effectiveness. Therefore, surgical staff should be sufficiently trained in wearing the PPE and powered air-purifying respirators, which may also reduce resource consumption [3].

In addition, the staff should decrease their exposure to symptomatic or asymptomatic individuals who present in the hospital even for unrelated ailments by remaining outside the facility and self-isolating to the extent possible [3]. Accordingly, it has been proposed that staff should monitor their body temperature twice a day and enter this information in web-based forms via their smartphones to identify symptomatic cases [7].

OR configuration is also a crucial issue. Reports from the United States and Singapore have proposed that each hospital should develop separate alternate surgical complexes, away from high-traffic areas, for patients with confirmed or suspected COVID-19 [3,7]. An anteroom or a taped off area should be used by the staff to wear the PPE and organize the materials and medication needed for each case. Only the necessary items for each surgery should be brought in the OR, and disposable caps and shoe covers should be discarded after their first use. Additionally, exiting the OR during surgery should be kept to a minimum. A staff worker could be present in the anteroom and provide the materials needed during the operation using a material exchange cart.

Moreover, special attention is needed in performing each operation technique. Specifically, electrocautery and dissection times should be limited to reduce surgical smoke. Likewise, the surgeons should be aware of any injury with a sharp implement. Moreover, it has been suggested that a 18-min air-exchange is needed to ensure clean OR air for the next patient, and each used device should undergo disinfection [7]. This additional time should also be considered by the surgeons and the anesthesiologists when scheduling the forthcoming operations.

Finally, the recovery and transportation of the patient to his/her room after surgery is crucial. Ideally, the patient should recover in the OR and, then transferred to an isolation room or in the intensive care unit through corridors that are retained clear of other patients [5].

Undoubtedly, the impact of COVID-19 in surgical care has been far-reaching, and the aforementioned measures could be considered exhaustive. However, surgery remains an indispensable part of health care that cannot be entirely abolished during the pandemic. Designing clear surgical strategies and informing all surgeons regarding the appropriate measures are necessary in the battle against COVID-19.

## Ethical approval

The need for ethical approval was waived as it was a commentary article, without involving participants.

## Sources of funding

There was no funding for this study.

## Author contribution

All authors were equally contributed in the writing of this manuscript.

## Registration of research studies

1. Name of the registry:

2. Unique Identifying number or registration ID:

3. Hyperlink to your specific registration (must be publicly accessible and will be checked):

## Guarantor

Ypatios Spanidis.

Department of Surgery, School of Medicine.

University of Patras, Patras, Greece.

Post Code: 26504.

Tel: +306980068997.

E-mail: akis.span@yahoo.gr.

## Consent

There was no need for informed consent as this study did not involve human subjects.

## Provenance and peer review

Not commissioned, Editor reviewed.

## Declaration of competing interest

The authors report no conflicts of interest.
